# 2-(1,2,3,4-Tetra­hydro­naphthalen-1-yl­idene)hydrazinecarbothio­amide

**DOI:** 10.1107/S1600536812033302

**Published:** 2012-07-28

**Authors:** Adriano Bof de Oliveira, Cecília Santos Silva, Bárbara Regina Santos Feitosa, Christian Näther, Inke Jess

**Affiliations:** a Departamento de Química, Universidade Federal de Sergipe, Av. Marechal Rondon s/n, Campus, 49100-000 São Cristóvão-SE, Brazil; b Institut für Anorganische Chemie, Christian-Albrechts-Universität zu Kiel, Max-Eyth Strasse 2, D-24118 Kiel, Germany

## Abstract

The mol­ecular structure of the title compound, C_11_H_13_N_3_S, is not planar: the maximum deviation from the mean plane of the non-H atoms is 0.521 (2) Å for an aliphatic C atom, which corresponds to an envelope conformation for the non-aromatic ring. The hydrazinecarbothio­amide substituent and the benzene ring have maximum deviations from the mean planes through the non-H atoms of 0.0288 (16) and 0.0124 (27) Å, respectively, and the dihedral angle between the two planes is 8.84 (13)°. In the crystal, mol­ecules are linked into chains along [1
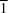
0] by pairs of N—H⋯S hydrogen bonds between mol­ecules related by centres of symmetry.

## Related literature

For the synthesis of the title compound and the pharmacological activity of ketonethio­semicrabazones, see: Thanigaimalai *et al.* (2011 ▶). For crystal structures of other thio­semicarbazone derivatives with pharmacological activity, see: Pederzolli *et al.* (2011 ▶); Bittencourt *et al.* (2012 ▶).
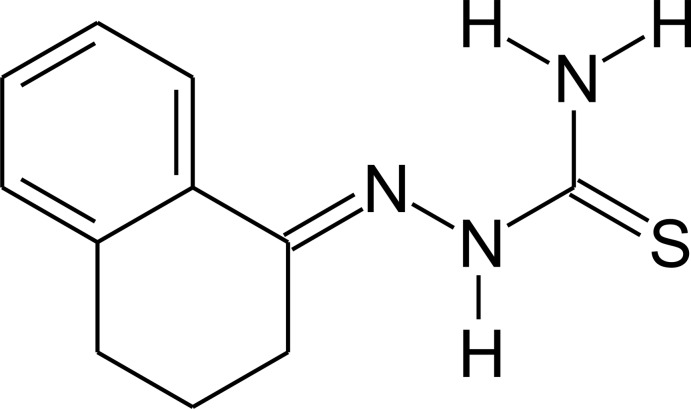


## Experimental

### 

#### Crystal data

C_11_H_13_N_3_S
*M* *_r_* = 219.30
Monoclinic, 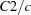

*a* = 15.4388 (11) Å
*b* = 5.5781 (3) Å
*c* = 26.338 (2) Å
β = 102.940 (6)°
*V* = 2210.6 (3) Å^3^
*Z* = 8
Mo *K*α radiation
μ = 0.26 mm^−1^
*T* = 293 K
0.3 × 0.2 × 0.2 mm

#### Data collection

Stoe IPDS-1 diffractometer
7673 measured reflections
2402 independent reflections
2019 reflections with *I* > 2σ(*I*)
*R*_int_ = 0.043

#### Refinement

*R*[*F* ^2^ > 2σ(*F* ^2^)] = 0.045
*wR*(*F* ^2^) = 0.126
*S* = 1.08
2402 reflections
136 parameters
H-atom parameters constrained
Δρ_max_ = 0.19 e Å^−3^
Δρ_min_ = −0.21 e Å^−3^

### 

Data collection: *X-AREA* (Stoe & Cie, 2008 ▶); cell refinement: *X-AREA*; data reduction: *X-RED32* (Stoe & Cie, 2008 ▶); program(s) used to solve structure: *SHELXS97* (Sheldrick, 2008 ▶); program(s) used to refine structure: *SHELXL97* (Sheldrick, 2008 ▶); molecular graphics: *XP* in *SHELXTL* (Sheldrick, 2008 ▶); software used to prepare material for publication: *publCIF* (Westrip, 2010 ▶).

## Supplementary Material

Crystal structure: contains datablock(s) I, global. DOI: 10.1107/S1600536812033302/fy2063sup1.cif

Structure factors: contains datablock(s) I. DOI: 10.1107/S1600536812033302/fy2063Isup2.hkl

Supplementary material file. DOI: 10.1107/S1600536812033302/fy2063Isup3.cml

Additional supplementary materials:  crystallographic information; 3D view; checkCIF report

## Figures and Tables

**Table 1:** Hydrogen-bond geometry (Å, °)

*D*—H⋯*A*	*D*—H	H⋯*A*	*D*⋯*A*	*D*—H⋯*A*
N2—H1*N*2⋯S1^i^	0.89	2.71	3.5606 (14)	161
N3—H1*N*3⋯S1^ii^	0.89	2.45	3.3351 (16)	171

Symmetry codes: (i) 
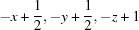
; (ii) 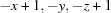.

**Fig. 1.**
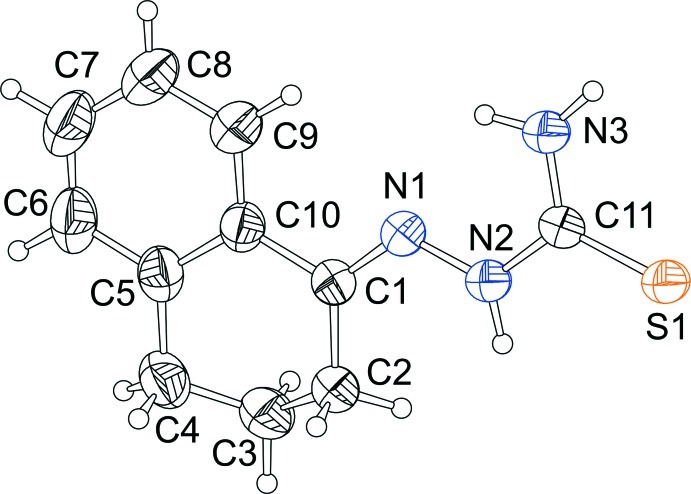
Molecular structure of the title compound with labeling and displacement ellipsoids drawn at the 40% probability level.

**Fig. 2.**
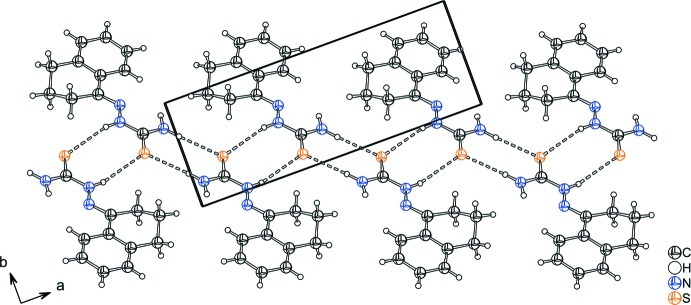
Crystal structure of the title compound with view along the crystallographic c axis, showing the N—H···S hydrogen bonding as dashed lines.

## supplementary crystallographic information

### Comment

Thiosemicarbazone derivatives have a wide range of pharmacological properties. For example, ketonethiosemicarbazones show pharmacological activity against melanogenesis in melanoma B16 cells (Thanigaimalai *et al.*, 2011). As part of our study on the synthesis of thiosemicarbazone derivatives, we report herein the crystal structure of 2-(3,4-dihydronaphthalen-1(*2H*)-ylidene)hydrazinecarbothioamide.

In the crystal structure of the title compound the maximum deviation from the least squares plane through all non-hydrogen atoms is 0.5205 (23) Å for C3, which is in agreement with the envelope conformation observed for the non-aromatic ring (Fig. 1).

The molecule shows an *trans* conformation for the atoms about the C1—N1/N1—N2/N2—C11 bonds. The mean deviations from the least squares planes for the N1/N2/C11/N3/S1 and C5/C6/C7/C8/C9/C10 fragments amount to 0.0288 (16) Å for N2 and 0.0124 (27) Å for C7, respectively, and the dihedral angle between the two planes is 8.84 (13)°. The *trans* conformation for the thiosemicarbazone fragment is also observed in other structures (Pederzolli *et al.*, 2011 and Bittencourt *et al.*, 2012).

The molecules are connected *via* centrosymmetric pairs of N—H···S hydrogen bonds, forming a one-dimensional H-bonded polymer along [1 -1 0] (Fig. 2 and Table 1).

### Experimental

All starting materials were commercially available and were used without further purification. The synthesis was adapted from a procedure reported previously (Thanigaimalai *et al.*, 2011). The hydrochloric acid catalyzed reaction of 1-tetralone (10 mmol) and thiosemicarbazide (10 mmol) in a 3:1 mixture of ethanol and water (100 ml) was refluxed for 7 h. After cooling and filtering, crystals suitable for X-ray diffraction were obtained by recrystallization from tetrahydrofurane.

### Refinement

All non-hydrogen atoms were refined anisotropically. C—H H atoms were positioned with idealized geometry and were refined isotropically, with *U*_iso_(H) = 1.2 *U*_eq_(C) using a riding model with C—H = 0.97 Å for aromatic and 0.93 Å for methylene H atoms. N—H H atoms were located in difference map, their bond lengths set to 0.89 Å and finally they were refined isotropically with *U*_iso_(H) = 1.2 *U*_eq_(N) using a riding model.

### Crystal data

**Table:**

C_11_H_13_N_3_S	*Z* = 8
*M**_r_* = 219.30	*F*(000) = 928
Monoclinic, *C*2/*c*	*D*_x_ = 1.318 Mg m^−^^3^
Hall symbol: -C 2yc	Mo *K*α radiation, λ = 0.71073 Å
*a* = 15.4388 (11) Å	µ = 0.26 mm^−^^1^
*b* = 5.5781 (3) Å	*T* = 293 K
*c* = 26.338 (2) Å	Block, yellow
β = 102.940 (6)°	0.3 × 0.2 × 0.2 mm
*V* = 2210.6 (3) Å^3^	

### Data collection

**Table:**

Stoe IPDS-1 diffractometer	2019 reflections with *I* > 2σ(*I*)
Radiation source: fine-focus sealed tube, Stoe IPDS-1	*R*_int_ = 0.043
Graphite monochromator	θ_max_ = 27.0°, θ_min_ = 3.4°
φ scans	*h* = −19→19
7673 measured reflections	*k* = −6→7
2402 independent reflections	*l* = −29→33

### Refinement

**Table:**

Refinement on *F*^2^	Primary atom site location: structure-invariant direct methods
Least-squares matrix: full	Secondary atom site location: difference Fourier map
*R*[*F*^2^ > 2σ(*F*^2^)] = 0.045	Hydrogen site location: inferred from neighbouring sites
*wR*(*F*^2^) = 0.126	H-atom parameters constrained
*S* = 1.08	*w* = 1/[σ^2^(*F*_o_^2^) + (0.0611*P*)^2^ + 0.6719*P*] where *P* = (*F*_o_^2^ + 2*F*_c_^2^)/3
2402 reflections	(Δ/σ)_max_ < 0.001
136 parameters	Δρ_max_ = 0.19 e Å^−^^3^
0 restraints	Δρ_min_ = −0.21 e Å^−^^3^

### Special details

**Table:**

Geometry. All e.s.d.'s (except the e.s.d. in the dihedral angle between two l.s. planes) are estimated using the full covariance matrix. The cell e.s.d.'s are taken into account individually in the estimation of e.s.d.'s in distances, angles and torsion angles; correlations between e.s.d.'s in cell parameters are only used when they are defined by crystal symmetry. An approximate (isotropic) treatment of cell e.s.d.'s is used for estimating e.s.d.'s involving l.s. planes.
Refinement. Refinement of *F*^2^ against ALL reflections. The weighted *R*-factor *wR* and goodness of fit *S* are based on *F*^2^, conventional *R*-factors *R* are based on *F*, with *F* set to zero for negative *F*^2^. The threshold expression of *F*^2^ > σ(*F*^2^) is used only for calculating *R*-factors(gt) *etc*. and is not relevant to the choice of reflections for refinement. *R*-factors based on *F*^2^ are statistically about twice as large as those based on *F*, and *R*- factors based on ALL data will be even larger.

### Fractional atomic coordinates and isotropic or equivalent isotropic displacement parameters (Å^2^)

**Table:**

	*x*	*y*	*z*	*U*_iso_*/*U*_eq_	
C1	0.29958 (11)	0.7852 (3)	0.39372 (7)	0.0547 (4)	
C2	0.21106 (12)	0.8320 (4)	0.40609 (8)	0.0651 (5)	
H2A	0.2187	0.9398	0.4357	0.078*	
H2B	0.1869	0.6825	0.4157	0.078*	
C3	0.14622 (13)	0.9414 (4)	0.36024 (9)	0.0748 (6)	
H3A	0.1336	0.8271	0.3318	0.090*	
H3B	0.0909	0.9779	0.3702	0.090*	
C4	0.18397 (16)	1.1667 (4)	0.34255 (10)	0.0803 (6)	
H4A	0.1438	1.2255	0.3113	0.096*	
H4B	0.1882	1.2883	0.3693	0.096*	
C5	0.27389 (14)	1.1280 (3)	0.33150 (7)	0.0647 (5)	
C6	0.30605 (19)	1.2810 (4)	0.29776 (9)	0.0837 (6)	
H6	0.2709	1.4078	0.2821	0.100*	
C7	0.3882 (2)	1.2473 (5)	0.28746 (10)	0.0920 (7)	
H7	0.4087	1.3528	0.2654	0.110*	
C8	0.43993 (17)	1.0605 (5)	0.30923 (10)	0.0881 (7)	
H8	0.4950	1.0357	0.3013	0.106*	
C9	0.41110 (15)	0.9082 (4)	0.34300 (9)	0.0761 (6)	
H9	0.4472	0.7820	0.3581	0.091*	
C10	0.32796 (12)	0.9407 (3)	0.35500 (7)	0.0583 (4)	
N1	0.35565 (10)	0.6249 (3)	0.41497 (6)	0.0566 (4)	
N2	0.33609 (9)	0.4805 (3)	0.45306 (6)	0.0580 (4)	
H1N2	0.2861	0.4818	0.4646	0.070*	
C11	0.39552 (11)	0.3092 (3)	0.47378 (7)	0.0540 (4)	
N3	0.46628 (10)	0.2886 (3)	0.45383 (7)	0.0680 (5)	
H1N3	0.5063	0.1731	0.4636	0.082*	
H2N3	0.4719	0.4075	0.4323	0.082*	
S1	0.37784 (3)	0.13624 (10)	0.52278 (2)	0.0675 (2)	

### Atomic displacement parameters (Å^2^)

**Table:**

	*U*^11^	*U*^22^	*U*^33^	*U*^12^	*U*^13^	*U*^23^
C1	0.0598 (9)	0.0488 (9)	0.0579 (9)	0.0039 (7)	0.0182 (7)	0.0021 (7)
C2	0.0643 (10)	0.0616 (11)	0.0741 (12)	0.0111 (8)	0.0254 (9)	0.0117 (9)
C3	0.0649 (11)	0.0767 (13)	0.0823 (14)	0.0139 (10)	0.0152 (10)	0.0063 (11)
C4	0.0913 (14)	0.0677 (13)	0.0816 (14)	0.0220 (11)	0.0186 (11)	0.0137 (11)
C5	0.0867 (13)	0.0505 (10)	0.0566 (10)	0.0006 (9)	0.0153 (9)	0.0002 (8)
C6	0.1232 (19)	0.0616 (12)	0.0656 (12)	−0.0012 (12)	0.0197 (12)	0.0117 (10)
C7	0.128 (2)	0.0829 (16)	0.0730 (14)	−0.0202 (15)	0.0384 (14)	0.0123 (12)
C8	0.0951 (16)	0.0975 (17)	0.0812 (15)	−0.0122 (14)	0.0396 (13)	0.0123 (13)
C9	0.0788 (13)	0.0780 (14)	0.0779 (13)	0.0020 (10)	0.0310 (11)	0.0133 (11)
C10	0.0706 (10)	0.0515 (9)	0.0546 (9)	−0.0013 (8)	0.0178 (8)	0.0023 (8)
N1	0.0617 (8)	0.0545 (8)	0.0578 (8)	0.0057 (6)	0.0226 (6)	0.0075 (7)
N2	0.0561 (8)	0.0595 (9)	0.0641 (8)	0.0119 (6)	0.0257 (7)	0.0127 (7)
C11	0.0531 (8)	0.0519 (9)	0.0604 (10)	0.0071 (7)	0.0199 (7)	0.0017 (7)
N3	0.0628 (9)	0.0686 (10)	0.0820 (11)	0.0186 (7)	0.0362 (8)	0.0212 (8)
S1	0.0622 (3)	0.0737 (4)	0.0741 (3)	0.0200 (2)	0.0316 (2)	0.0242 (2)

### Geometric parameters (Å, º)

**Table:**

C1—N1	1.282 (2)	C6—H6	0.9300
C1—C10	1.478 (2)	C7—C8	1.359 (4)
C1—C2	1.497 (2)	C7—H7	0.9300
C2—C3	1.514 (3)	C8—C9	1.374 (3)
C2—H2A	0.9700	C8—H8	0.9300
C2—H2B	0.9700	C9—C10	1.401 (3)
C3—C4	1.503 (3)	C9—H9	0.9300
C3—H3A	0.9700	N1—N2	1.3722 (19)
C3—H3B	0.9700	N2—C11	1.352 (2)
C4—C5	1.497 (3)	N2—H1N2	0.8899
C4—H4A	0.9700	C11—N3	1.319 (2)
C4—H4B	0.9700	C11—S1	1.6818 (17)
C5—C10	1.393 (3)	N3—H1N3	0.8900
C5—C6	1.401 (3)	N3—H2N3	0.8900
C6—C7	1.368 (4)		
			
N1—C1—C10	115.77 (15)	C7—C6—H6	119.4
N1—C1—C2	125.91 (16)	C5—C6—H6	119.4
C10—C1—C2	118.28 (15)	C8—C7—C6	120.3 (2)
C1—C2—C3	111.69 (16)	C8—C7—H7	119.8
C1—C2—H2A	109.3	C6—C7—H7	119.8
C3—C2—H2A	109.3	C7—C8—C9	120.1 (2)
C1—C2—H2B	109.3	C7—C8—H8	119.9
C3—C2—H2B	109.3	C9—C8—H8	119.9
H2A—C2—H2B	107.9	C8—C9—C10	120.8 (2)
C4—C3—C2	110.56 (19)	C8—C9—H9	119.6
C4—C3—H3A	109.5	C10—C9—H9	119.6
C2—C3—H3A	109.5	C5—C10—C9	119.00 (18)
C4—C3—H3B	109.5	C5—C10—C1	120.42 (16)
C2—C3—H3B	109.5	C9—C10—C1	120.53 (17)
H3A—C3—H3B	108.1	C1—N1—N2	119.39 (14)
C5—C4—C3	112.39 (17)	C11—N2—N1	118.00 (13)
C5—C4—H4A	109.1	C11—N2—H1N2	115.5
C3—C4—H4A	109.1	N1—N2—H1N2	126.4
C5—C4—H4B	109.1	N3—C11—N2	116.66 (15)
C3—C4—H4B	109.1	N3—C11—S1	123.15 (13)
H4A—C4—H4B	107.9	N2—C11—S1	120.19 (12)
C10—C5—C6	118.5 (2)	C11—N3—H1N3	122.2
C10—C5—C4	120.74 (17)	C11—N3—H2N3	113.4
C6—C5—C4	120.78 (19)	H1N3—N3—H2N3	124.3
C7—C6—C5	121.2 (2)		

### Hydrogen-bond geometry (Å, º)

**Table:**

*D*—H···*A*	*D*—H	H···*A*	*D*···*A*	*D*—H···*A*
N2—H1*N*2···S1^i^	0.89	2.71	3.5606 (14)	161
N3—H1*N*3···S1^ii^	0.89	2.45	3.3351 (16)	171

Symmetry codes: (i) −*x*+1/2, −*y*+1/2, −*z*+1; (ii) −*x*+1, −*y*, −*z*+1.
